# A two-stage improved variable neighborhood search-sine cosine algorithm for the multi-row layout problem with safety consideration

**DOI:** 10.1038/s41598-025-25551-x

**Published:** 2025-11-14

**Authors:** Achmad Pratama Rifai, Wangi Pandan Sari

**Affiliations:** Department of Mechanical and Industrial Engineering, Faculty of Engineering, Universitas Gadjah Mada, Yogyakarta, Indonesia

**Keywords:** Multi-row layout problem, Safe distance, Variable neighborhood search, Sine cosine algorithm, Engineering, Mathematics and computing

## Abstract

The multi-row layout problem (MRLP) involves arranging machines of varying sizes across multiple rows to minimize material handling costs. It is a significant design problem that frequently arises in practical situations. Some industrial settings require safety regulations to ensure a minimum distance between machines. However, existing studies on MRLP generally disregard the clearance between adjacent machines or solely take into account the minimum clearance. In this study, we address the issue by incorporating a safety factor into the MRLP and proposing a two-stage improved variable neighborhood with search-sine cosine algorithm (IVNS + SCA). The first stage involves an improved variable neighborhood search (IVNS) to determine machine placement on all rows. In the second stage, a sine-cosine algorithm (SCA) is presented to fine-tune the machines placement. The effectiveness and efficiency of the proposed algorithm are demonstrated through extensive computational testing at various levels of complexity and benchmarked against other heuristics algorithms. The proposed IVNS-SCA achieved an average improvement of 0.9–5.3% over the benchmark metaheuristics, with notably higher gains in large-sized instances.

## Introduction

The layout problem is a well-known combinatorial optimization problem that has been extensively studied in various fields, including manufacturing, logistics, and facility planning. A well-designed layout can have a significant impact on the efficiency and productivity of the manufacturing process, as well as on the safety of workers and the quality of the products being produced. In general, the facility layout is designed such that it can minimize the material handling cost (MHC), which is a non-value-added cost. MHC can contribute to about 20–50% of the total operating cost^1^ and by addressing layout problems, the cost can be reduced by at least 10–30%^2^.

In the single-row layout problem (SRLP), facilities are arranged along one side of the material handling path. On the other hand, multi-row layout problem (MRLP) involves several rows of facilities, of which the arrangements are laid on both sides of the material handling path. In practicality where numerous actual facilities place their machines (or rooms) on both sides of a corridor, the MRLP often addresses flow structure in a more efficient and realistic manner than SRLP. However, discussions on MRLP are limited in literature, especially in comparison to SRLP. Further, to the best of our knowledge, there is no prior research that has investigated the safety factor in MRLP. Most existing studies have only considered extra clearance between adjacent machines. Whereas the safety factor, representing the minimum distance between machines or workstations, is crucial for facility layout arrangements. Neglecting safety factors can lead to hazardous conditions such as fires, explosions, or other accidents, which could cause physical harm to workers, damage to equipment, and production downtime^3^. Safety factors also ensure environmental protection by preventing pollution, noise, and vibration, which could be harmful to the surrounding environment and people. Henceforth, it is important to consider safety distance between machines or workstations in finding the most efficient arrangement of facilities. In this study, we aim to bridge the research gap by incorporating safety distance consideration in the proposed mathematical formulation for the MRLP.

The objective of this study is twofold: firstly, to minimize MHC, and secondly, to integrate the consideration of the minimum safe distance between machines. The latter objective is achieved by minimizing the penalty incurred when the distance between the machines falls below the pre-defined recommended distance. The mathematical formulation for the MRLP is adopted from^4,5^. The proposed solution in this study adopts two-stage heuristics previously discussed in^6^. The first stage involves using an IVNS method to determine the optimal placement of machines in rows. To further improve the solution, an SCA is introduced to adjust the starting point of machines in the shorter row. The method is further referred to as improved variable neighborhood with search-sine cosine algorithm (IVNS + SCA).

The contributions of this study are threefold:

- First, this study is the first, to the best of our knowledge, to incorporate safety factors and recommended safe distances between machines in the MRLP. Previous works mainly considered only mutual interferences between adjacent machines, which results in merely setting minimum clearances.
- Second, we design the objective function to minimize the total material flow cost based on the actual travel distance of in-floor material handling equipment. This reflects practical industrial conditions, where equipment ranges from manual trolleys to fully automated systems, making the proposed model more applicable to real-world manufacturing environments.
- Third, we propose a two-stage heuristic approach tailored for industrial-scale MRLP with safety considerations. In Stage 1, the Improved Variable Neighborhood Search (IVNS) determines the machine sequence in each row. In Stage 2, the Sine Cosine Algorithm (SCA) fine-tunes the boundary clearance and the starting point of each row, ensuring both cost efficiency and safety compliance.

The remainder of this paper is structured as follows. Section 2 presents a literature review on the MRLP. The formulation of the MRLP with safety considerations is discussed in Sect. 3 whereas the proposed heuristic for the MRLP is detailed in Sect. 4. Section 5 thoroughly discusses numerical experiments. The paper concludes with Sect. 6, which presents the study’s conclusions and offers suggestions for future research.

## Literature review

The layout configuration and material handling system are mutually dependent on each other. The type of material handling system design impacts the layout configuration^7^ and vice versa^8^. Depending on the types of material handling system employed, the layout configurations can be categorized in terms of row numbers, i.e., single-row (SRLP), double-row (DRLP), or multi-row (MRLP)^9^. For a comprehensive review on facility layout problems, refer to^9–11^. The SRLP is concerned with finding the most efficient arrangement of *n* machines along one side of the material handling path. It is commonly used and popular in practice due to its simplicity and efficient workflow. Various solutions have been proposed for the SRLP including using exact methods^12,13^ and heuristics^14,15^, and metaheuristics^16,17^. The DRLP is an extension of the SRLP in which *n* machines can be placed on both sides of the material handling path. Chung and Tanchoco^18^ extended the SRLP into DRLP and solved up to 10 machines using Mixed integer Programming (MIP) approach. A more efficient model for DRLP was proposed with improved performance^19–21^. More recently, Amaral^22^ proposed a heuristic approach where two-phase algorithms were employed to obtain good solution with the number of instances up to 50 machines.

As the facilities arrangement for the MRLP are on both sides of a corridor, the optimization problem of MRLP is more complex than SRLP. Further, the topic has received significantly less attention in the literature^9^. Metaheuristic approaches such as Genetic Algorithm (GA) have been proposed to address MRLP aiming to minimize the MHC in manufacturing system^23,24^. Vitayasak et al.^25^ developed a GA-based tool for machine layout design with consideration of dynamic demand and machine maintenance planning to minimize material flow distance. Variable neighborhood search (VNS) has also been applied to find optimal facility arrangements in MRLPs without inter-facility spacing^26^. The neighborhood structure is produced by two different moves, i.e., exchanging two facilities and inserting a facility in a different position of the layout. Integration of machine layout problem and cell formation using heuristic method has yielded the most efficient solution for MRLP^27^. The authors introduce three design features, i.e., machine layout within the cells, machine layout on the planar area, and the distance between rows. They also proposed three lower bounds for the integrated problem and selected the tightest one to evaluate the heuristics’ solution, showing the method’s effectiveness in solving medium and large-scale problems. To address bi-objective optimization in MRLP, a heuristic method was developed to minimize both flow distance and layout area^28^. Additionally, a harmony search algorithm was employed to identify the optimal sequence of machines for multi-row arrangements, simultaneously minimizing both objectives. The validation of the method was tested on SRLP presented in Vitayasak and Pongcharoen’s work^28^. Another study by Zuo et al.^29^ proposed a three-stage approach for minimizing MHC in MRLP. First, a Monte Carlo heuristic is used to find the effective way of laying machines in multiple rows. Second, a linear program is used to identify the optimal exact location of machines. The last one is to utilize an exchange heuristic to reassign material flows among parallel machines in different machine groups. This heuristic helps to optimize the material flow and minimize the handling cost of products.

Clearance between adjacent machines is a crucial aspect of MRLP, as it impacts material handling, interference prevention, and safety. Gen et al.^23^ introduced the concept of fuzzy clearance between two adjacent machines for MRLP. On the other hand, a model was developed to solve MRLP by minimizing clearance between two adjacent machines^30^. This is an extended study of similar work conducted for DRLP. Another study explored shared clearances between machines to improve machine layout and material handling cost. The authors presented the problem with a MILP formulation and solved it using a hybrid approach comprising Tabu search and heuristic rules^31^. While some studies as previously mentioned considered the least or minimum clearance to MRLP, increasing the clearance between adjacent machines can potentially reduce production costs, provide better material handling and safety. However, studies on MRLP that incorporate extra clearance have been limited. Zuo et al. considered extra clearance between adjacent machines to solve MRLP with parallel machines^29^. A more recent study on MRLP incorporated extra clearance to optimize both material handling costs and layout area^32^. Further, most prior studies on MRLP have overlooked the incorporation of safety measures in their model. Such measures are crucial for maintaining a safe working environment by enforcing safety distance between machines.

The inclusion of safety factor in facility layout problems was previously discussed in an SRLP proposed by Ou-Yang and Utamima^33^. They proposed an enhanced SRLP with the objective of minimizing the total distances between all facility pairs, incorporating penalty costs for solutions that violated safety constraint. They employed a hybrid approach combining EDA, PSO, and TS to solve the problem. A more recent study on SRLP^34^ incorporated safety measures into the proposed model and utilized a hybrid approach consisting of GA and TS. This model introduced risk costs in the total layout cost for two machines based on their safety relation and proximity. If the machines shared clearance despite high safety risks, they were subjected to risk penalty cost. In our previous work, we also explored the inclusion of safety factors in DRLP and introduced a model that imposed penalties on the objective function when the distance between machines fell below the pre-defined recommended safety distance^6^. To the best of our knowledge, no previous study on MRLP has incorporated safety factors.

This study builds upon the model proposed by Amaral et al.^4^ and Fischer et al.^5^ by introducing a penalty mechanism in the objective function. The penalty is applied when the distance between machines is below under the pre-defined recommended safety distance, which is contingent on various factors including machine interferences (due to noise, vibration, or heat), regulatory compliance, and minimum space for material handling, and fire safety considerations, which are specific to the type of machinery involved. The varying severity of those factors may influence the recommendation of the safety distance. Hence, employing a penalty function in the objective function offers greater flexibility for decision makers to determine the appropriate safety distance. We propose a VNS based two-stage metaheuristic referred to as IVNS + SCA. VNS, firstly introduced by Mladenovic and Hansen^35^, is a classical metaheuristic approach used to solve complex combinatorial optimization problems. Its efficacy has been demonstrated in various studies on facility layout problems, including SRLP^36–38^, DRLP^6,39^, and facilities with unequal area^40,41^. Whereas SCA is a relatively new algorithm proposed by Mirjalili in 2016 to solve optimization problems^42^. Due to its flexibility and simplicity, the SCA method has attracted much attention from researchers and been successfully applied to solve various optimization problems, such as the scheduling^43–45^, traveling salesman problem^46^, and image processing^47,48^.

Our study proposes a two-stage heuristic solution approach for the MRLP with safety considerations for industrial-scale cases. The first stage uses IVNS to determine machine sequences in each row, whilst the second stage employs SCA to identify the boundary clearance and starting point of each row. This approach addresses the gap in the literature by demonstrating the viability of incorporating both metaheuristic algorithms and safety factors into MRLP. For an overview of facility layout problems based on materials handling configurations, refer to Table 1.

**Table 1:** Overview of facility layout problems based on material handling configurations.

References	Material handling configurations	Solution category	Solution approach	Safety considerations
^12^	SRLP	Exact	SDP	No
^13^	SRLP	Math programming, exact	MILP - branch and cut algorithms	No
^14^	SRLP	Heuristic	Best insertion (BI) and iterative BI	No
^15^	SRLP	Heuristic	Improvement heuristic based on Lin–Kernighan algorithm	No
^16^	SRLP	Heuristic	GA	No
^17^	SRLP	Heuristic	TA (2-opt and insertion neighborhood)	No
^18^	DRLP	Math programming, heuristic	MIP, comparison of several heuristics	No
^19^	DRLP	Math programming	Improved MIP	No
^20^	DRLP	Math programming	Improved MIP	No
^21^	DRLP	Math programming	Improved MIP	No
^22^	DRLP	Heuristic	Several improvement heuristics	No
^23^	MRLP	Heuristic	GA	No
^26^	MRLP	Heuristic	VNS	No
^27^	MRLP	Math programming, heuristic	MINLP/decomposition	No
^28^	MRLP	Heuristic	Harmony search algorithm	No
^29^	MRLP	Heuristic	Improvement heuristic based on Monte Carlo, Linear Program, and Exchange Heuristic	No
^30^	MRLP	Math programming, exact	SDP	No
^31^	MRLP	Math programming, heuristic	MILP, TS	No
^32^	MRLP	Math programming, heuristic	MIP, mGRASP	No
^33^	SRLP	Heuristic	A hybrid algorithm based on EDA, PSO, and TS	Yes
^34^	SRLP	Heuristic	Improved GA & TS	Yes
^36^	SRLP	Heuristic	VNS and ACO	No
^37^	SRLP	Heuristic	LS-VNS	No
^38^	SRLP	Heuristic	LS-VNS	No
^6^	DRLP	Heuristic	IVNS-SCA	Yes
^39^	DRLP	Math programming	MIP	Yes
This study	MRLP	Heuristic	IVNS-SCA	Yes

## Problem formulation

This section explains the model for the multi-row layout problem with safety consideration. In this study, we expand the MRLP to encompass real-world scenarios where individual machines possess specific recommended safety distances, and each row is limited by dimensional constraints. When organizing the layout of machines, decision-makers frequently encounter the necessity to adhere to safety regulations, like maintaining minimum distances between machines or workstations. This element remains unaccounted for in the initial MRLP formulation, despite the potential hazards arising from disregarding these constraints, including risks of fire, explosions, noise, vibration, and pollution.

**Fig. 1:**
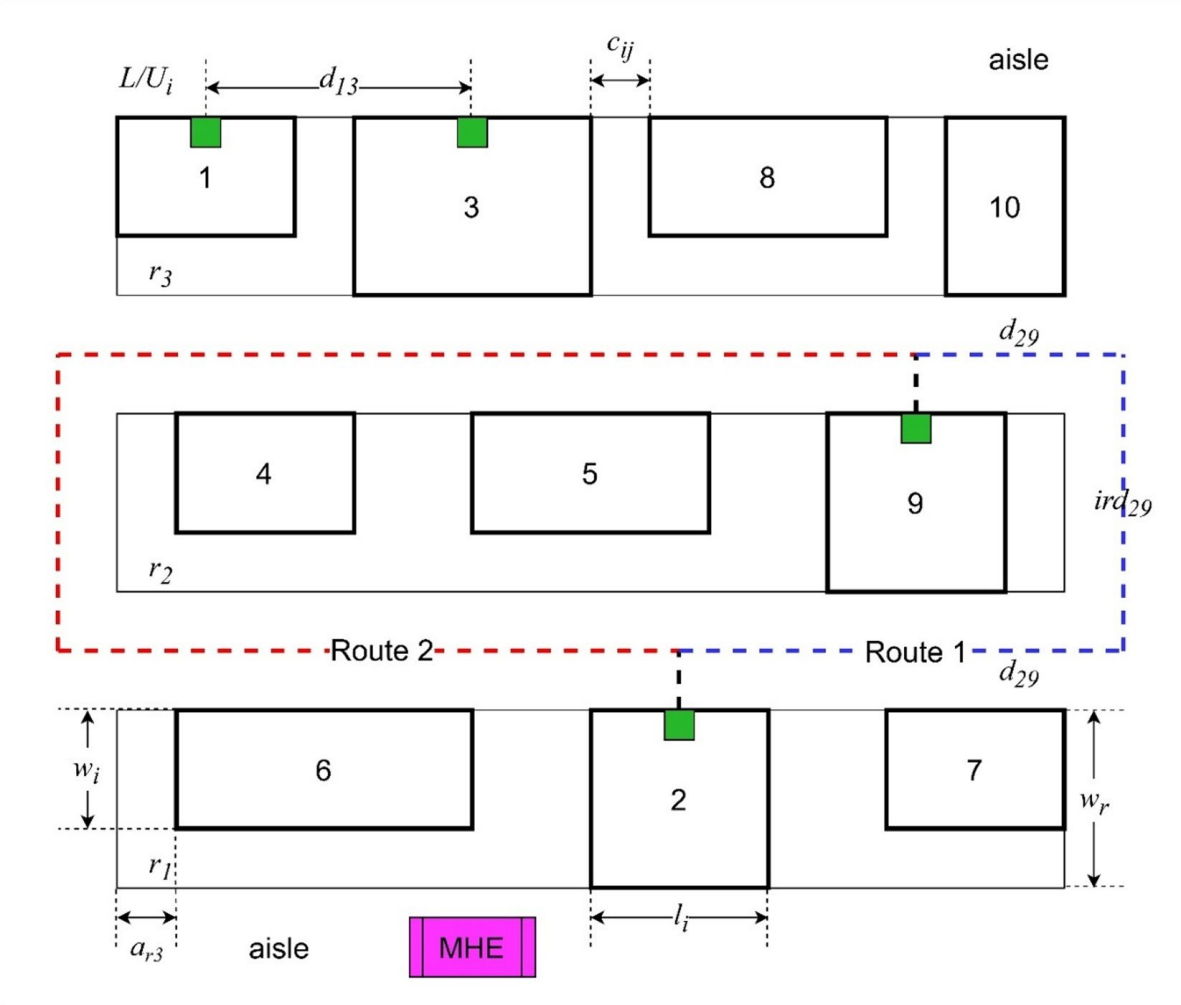
Example of a multi-row layout.

The multi-row layout consists of several rows of machine, in which two adjacent rows are separated by an aisle/corridor. The type of material handling equipment (MHE) used in this layout are two-way in-floor MHE, such as automated guided vehicle (AGV), pallet jack, and forklift. The aisles provide access for the MHE movement. Each row is served by an aisle section. There are two additional aisle sections, each located at the left and right ends of the layout that serve inter-row movements. Figure 1 presents an example of multi-row layout discussed in this study with $\:n=10$ and $\:m=3$.

In this layout, there are three rows: $\:{r}_{1}$, $\:{r}_{2}$, and $\:{r}_{3}$. The machine sequence on $\:{r}_{1}$, $\:{r}_{2}$, $\:{r}_{3}$ are {1, 3, 8,10}, {4, 5, 9}, and {6, 2, 7}, respectively. Each machine has a fixed length $\:{l}_{i}$. Although the machines may have different widths, the individual values are negligible since each row must have a fixed width. The row width $\:{w}_{r}$ is determined by the member machine which has the largest width $\:{w}_{r}=\underset{i\in\:{N}_{r}}{\text{max}}{w}_{i}$, in which $\:{N}_{r}$ is the set of machines belonging to row $\:r$.

The loading/unloading ports $\:{L/U}_{i}$ are located at the middle of each machine on the side which faces the corresponding aisle. As such, each machine only has a single loading/unloading port. There is a clearance between two machines $\:{c}_{ij}$, which is calculated as the distance between adjacent sides of the two machines.

The distance between any two machines, consisting of horizontal distance $\:{d}_{ij}$ and inter-row distance $\:ir{d}_{ij}$, is the actual distance travelled by the MHE to move the materials between the machines’ loading/unloading ports. The intra-row movements (material movement between two machines in the same row) only pass through the aisle section facing these two machines. However, in the inter-row movements (material movement between two machines in different rows), the MHE must pass through three aisle sections: one aisle section faced by the first machine, one aisle section faced by the second machine, and one additional aisle section for inter-row movement. As such, there are two routes for this type of movement, one route using right-end additional aisle section and another route using left-end additional aisle section as exemplified in Fig. 1 as route 1 and route 2, respectively. The selected route between the two routes must be the route that yields minimum distance. In the example, since the length of route 1 is less than the length of route 2, hence $\:{d}_{29}+ir{d}_{29}$ = length of route 1.

The consideration of the safety layout factor in this problem is represented as the recommended minimum distance *ϕ* between a pair of machines that must be fulfilled. As such, the closest distance between the two machines must be greater than or equal to their recommended minimum distance. For example, in Fig. 1, if $\:{d}_{13}=10,{ird}_{13}=0$ (since both machines are in the same row), and $\:{\varphi\:}_{13}=15$, then the placement of these two machines violates the safety considerations. Hence, in the mathematical model, penalty $\:{f}_{2}$ will be given.

The MRLP concerned in this study consist of two subproblems: determining the machine sequence on each row and determining the length of boundary clearance $\:a$ of each row. The boundary clearance is the clearance between the left side of the leftmost machine in each row and the layout boundary (Wan et al. 2022). For example, in Fig. 1, the boundary clearance of the third row is indicated as $\:{a}_{r3}$. In other words, the boundary clearance determines the starting point of each row. Noted that in the row with longest length, the boundary clearance is zero, hence the starting point of this row is always at point zero in *x*-axis.

Further, several assumptions were made for the model: (i) each machine/workstation is rectangular with fixed width and length dimensions, denoted as $\:{w}_{i}\:$and $\:{l}_{i}$, respectively, (ii) the width of the aisle/corridor is negligible, (iii) the product flows follow symmetric matrices, (iv) the distance between machines is calculated as the distance between their L/U ports (the center point), (v) the recommended minimum distance *ϕ* is outlined, which is a symmetric matrix. The sets, parameters, and decision variables of MRLP are listed below:

**Table:** 

Sets and indices	
$\:N$	= set of machines $\:\{1,\dots\:,n\}$
$\:i$	= index of machine $\:i\in\:N$
$\:R$	= set of rows $\:\{\text{1,2},\dots\:,m\}$,
$\:r$	= index of row $\:r\in\:R$
Parameters	
$\:n$	= number of machines
$\:m$	= number of available rows
$\:{l}_{i}$	= length of machine $\:i\in\:N$
$\:L$	= total length of all machines, $\:L=\sum\:_{i\in\:N}{l}_{i}$
$\:{w}_{i}$	= width of machine $\:i\in\:N$
$\:\beta\:$	= penalty factor
$\:{c}_{ij}$	= clearance between adjacent machines
$\:{cf}_{ij}$	= amount of material flow between machine $\:i$ and machine $\:j$
$\:{\varphi\:}_{ij}$	= recommended safe distance between machines $\:i$ and $\:j$
Variables	
$\:\widehat{R}$	= the total number of rows used in the layout, in which $\:{N}_{r}=\varnothing\:$
$\:{d}_{ij}$	= horizontal distance between the center of machines $\:i$ and $\:j$
$\:{ird}_{ij}$	= inter-row distance between machines $\:i$ and $\:j$
$\:{u}_{ikj}$	= Binary variable to indicate in which row a machine belongs to, 1 if machine $\:k$ is placed between machines $\:i$ and $\:j$ in the same row, and 0 otherwise. Consecutively, if the value is 1, this variable also indicates that machine $\:i$ is placed to the left of machine $\:j$.

Then, the following mixed-integer linear programming (MILP) formulation is proposed. The mathematical model is adopted from Amaral et al.^4^ and Fischer et al.^5^ in which we introduce novel objective functions and several constraints to comply the concerned problem.

1 $$\:\text{min}({f}_{1}+{f}_{2})$$

1a $$\:{f}_{1}=\sum\:_{i=1}^{n-1}\sum\:_{j=i+1}^{n}{cf}_{ij}{(d}_{ij}+{ird}_{ij})$$

1b $$\:{f}_{2}=\sum\:_{i=1}^{n-1}\sum\:_{j=i+1}^{n}\beta\:\times\:{Z}_{ij}$$

subject to:

2 $$\:{u}_{ijk}+{u}_{ikj}+{u}_{jik}=1\:\:\:\forall\:\:r\in\:R;i,j,k\in\:{N}_{r},\:i<j<k$$

3 $$\:{u}_{ihj}+{u}_{ihk}+{u}_{jhk}\le\:2\:\:\:\forall\:\:r\in\:R;i,j,k,h\in\:{N}_{r},\:i<j<k$$

4 $$\:{-u}_{ihj}+{u}_{ihk}+{u}_{jhk}\ge\:0\:\:\:\forall\:\:r\in\:R;i,j,k,h\in\:{N}_{r},\:i<j<k$$

5 $$\:{u}_{ihj}-{u}_{ihk}+{u}_{jhk}\ge\:0\:\:\:\forall\:\:r\in\:R;i,j,k,h\in\:{N}_{r},\:i<j<k$$

6 $$\:{u}_{ihj}+{u}_{ihk}-{u}_{jhk}\ge\:0\:\:\:\forall\:\:r\in\:R;i,j,k,h\in\:{N}_{r},\:i<j<k$$

7 $$\:{u}_{\left(n+1\right)i\left(n+2\right)}=1\:\:\:\forall\:\:i\in\:N$$

8 $$\:{u}_{i\left(n+1\right)j}=0\:\:\:\:\forall\:\:r\in\:R;i,j\in\:{N}_{r}\cup\:\:\{n\:+\:2\},\:i<j$$

9 $$\:{u}_{i\left(n+2\right)j}=0\:\:\:\forall\:\:r\in\:R;i,j\in\:{N}_{r}\cup\:\:\{n\:+\:1\},\:i<j$$

10 $$\:{u}_{ji\left(n+1\right)}={u}_{ij\left(n+2\right)}\:\:\:\forall\:\:r\in\:R;i,j\in\:{N}_{r},\:i\ne\:j$$

11 $$\:{d}_{j(n+1)}-{d}_{i\left(n+1\right)}\ge\:L\left({u}_{ji\left(n+1\right)}-1\right)+\frac{{l}_{i}+lj}{2}\:\:\:\forall\:\:r\in\:R;i,j\in\:{N}_{r},\:i\ne\:j$$

12 $$\:{d}_{i\left(n+1\right)}\ge\:\frac{{l}_{i}}{2}\:\:\:\forall\:\:i\in\:N$$

13 $$\:{d}_{ij}\ge\:{d}_{i\left(n+1\right)}-{d}_{j\left(n+1\right)}\:\:\:\forall\:\:r,o\in\:R;i\in\:{N}_{r},j\in\:{N}_{o},\:i<j$$

14 $$\:{d}_{ij}\ge\:{d}_{j\left(n+1\right)}-{d}_{i\left(n+1\right)}\:\:\:\forall\:\:r,o\in\:R;i\in\:{N}_{r},j\in\:{N}_{o},\:i<j$$

15 $$\:{d}_{ij}={d}_{i\left(n+1\right)}+{d}_{j\left(n+1\right)}\:\:\:\forall\:\:r,o\in\:R;i\in\:{N}_{r},j\in\:{N}_{o},\:i<j$$

$$\:{d}_{ij}+{d}_{jk}\ge\:{d}_{ik}$$

$$\:{d}_{ik}+{d}_{jk}\ge\:{d}_{ij}$$.

$$\:{d}_{ij}+{d}_{ik}\ge\:{d}_{jk}\quad \:\forall\:i,j,k\in\:{N}_{r},\:i<k$$

16 $$\:\text{m}\text{a}\text{x}\left\{\right|{r}_{s}-{r}_{t}|:s,t\in\:\left\{i,j,k\right\}\}\ge\:1$$

17 $$\:{d}_{i(n+1)}\ge\:\sum\:_{k\in\:{N}_{r}\backslash\:\left\{i\right\}}{l}_{k}{u}_{ik(n+1)}+\frac{{l}_{i}}{2}\:\:\:\forall\:\:r\in\:R;i\in\:{N}_{r}$$

18 $$\:{d}_{ij}\ge\:\sum\:_{k\in\:{N}_{r}\backslash\:\{i,j\}}{l}_{k}{u}_{ikj}+\frac{{l}_{i}+lj}{2}\:\:\:\forall\:\:r\in\:R;i,j\in\:{N}_{r},\:i<j$$

19 $$\:{ird}_{ij}=\left(\underset{i\in\:N}{{max}}{w}_{i}\right)|{r}_{i}-{r}_{j}|\:\:\:\forall\:\:r\in\:R;i,j\in\:{N}_{r},\:i<j$$

20 $$\:{x}_{ij}\ge\:{d}_{ij}\:\:\:\forall\:\:i,j\in\:N$$

21 $$\:{x}_{ij}\ge\:{ird}_{ij}\:\:\:\forall\:\:i,j\in\:N$$

22 $$\:{Z}_{ij}\ge\:0\:\:\:\forall\:\:i,j\in\:N$$

23 $$\:{Z}_{ij}\ge\:{\varphi\:}_{ij}-{x}_{ij}\:\:\:\forall\:\:i,j\in\:N$$

24 $$\:{u}_{ijk}\in\:\left[\text{0,1}\right]\:\:\:\forall\:\:i,j,k\in\:{N}_{r},\:i<k$$

25 $$\:{d}_{ij},{ird}_{ij}\le\:0]\:\:\:\forall\:\:i,j\in\:N\cup\:\:\{n\:+\:1\},\:i<j$$

The goal of objective function (1a) is to minimize the cost associated with handling materials among machines. Objective function (1b) aims to minimize the penalty incurred when the direct distance, either horizontal distance ($\:{d}_{ij}$) or inter-row distance ($\:{ird}_{ij})\:$between machines *i* and *j* is less than the recommended distance ($\:{\varphi\:}_{ij}$) that is predetermined. The coefficient β controls the relative importance of safety versus flow cost, with higher values assigning stronger penalties to safety distance violations, and vice versa. The auxiliary variable $\:{Z}_{ij}$ indicates the differences between the direct distance and the recommended distance if $\:{\varphi\:}_{ij}$ is larger, 0 otherwise.

Constraints and inequalities (2–6) express that the machines in each row do not overlap and that they correspond to some permutation in each row. These constraints are adopted from the model by Amaral^4^ and Fischer et al.^5^. As such, if machines $\:i,j,k\in\:{N}_{r}$ are placed in the same row, exactly one of them lies in between other machines and certain transitivity rules have to be satisfied. Subsequently, Constraints (7–10) ensure that in each row, all machines are placed between the dummy machines $\:n+1$ (the left border of the layout) and $\:n+2$ (the right border in each row).

Constraints (11–12) ensure that a minimal distance of $\:\frac{{l}_{i}+lj}{2}$ between machines $\:i,j\in\:{N}_{r}\cup\:\:\{n\:+\:1\}$ assigned to the same row. These betweenness constraints guarantee a workable arrangement of machines, positioned between departments $\:n\:+\:1$ and $\:n\:+\:2$, within each row. By incorporating dummy departments and their respective betweenness variables, we enable the indirect utilization of ordering variables (specific betweenness variables) and positional variables (specific distance variables). This approach enhances our ability to optimize the arrangement of machines, ensuring efficient operations within the specified departments.

Constraints (13–14) are special triangle inequalities that bound the distances between the departments. Additionally, we can break some symmetry by setting $\:{u}_{ij\left(n+1\right)}$= 0 for some fixed pair $\:\left|\left\{i\:,\:j\:\right\}\right|=2$ and $\:i\:,\:j\in\:{N}_{r}$. Constraint (15) provides the distances between machines $\:i,\:j\in\:N,\:i\ne\:j$, lying in different rows $\:{r}_{i}\ne\:{r}_{j}$ are determined by summing up the distances of each of the two machines to the left border, i.e., to $\:n+\:1$. Constraint (16) are the classic triangle inequalities, which are used for machines in the same rows or in subsequent neighbouring rows. In addition, Constraints (17–18) bounded the position of each department $\:i,\:j\in\:N$, represented by the distance $\:{d}_{i(n+1)}$. We can leverage the power of the betweenness model as outlined in Amaral et al.^4^ for machines within the same row. Moreover, utilizing the triangle inequalities, we can indirectly apply this model to machines in different rows that are interconnected through machine $\:n\:+\:1$, which serves as the left border. This strategic approach allows us to optimize operations across various rows, enhancing overall efficiency in the interconnected machines.

In this model, we propose inter-row distance $\:{ird}_{ij}$, formulated in Constraint (19). This variable calculates the distance travelled to move from one row to other rows through the left or right sides aisles, presented as vertical distance in Fig. 1. Here, we assume that the aisle width is neglected. Hence, the inter-row distance is solely influenced by the row width, which in turn is determined by the machine with largest width. Constraints (20–23) are for the linearization of auxiliary variable $\:{Z}_{ij}$ calculation $\:{Z}_{ij}=\text{m}\text{a}\text{x}(0,{\varphi\:}_{ij}-{\text{m}\text{a}\text{x}(d}_{ij},{ird}_{ij}))$. Lastly, Constraint (24) enforces the binary nature of the decision variables $\:{u}_{ijk}$, and Constraint (25) is non-negativity constraint for variables $\:{d}_{ij}$ and $\:{ird}_{ij}$.

## Proposed method

**Fig. 2:**
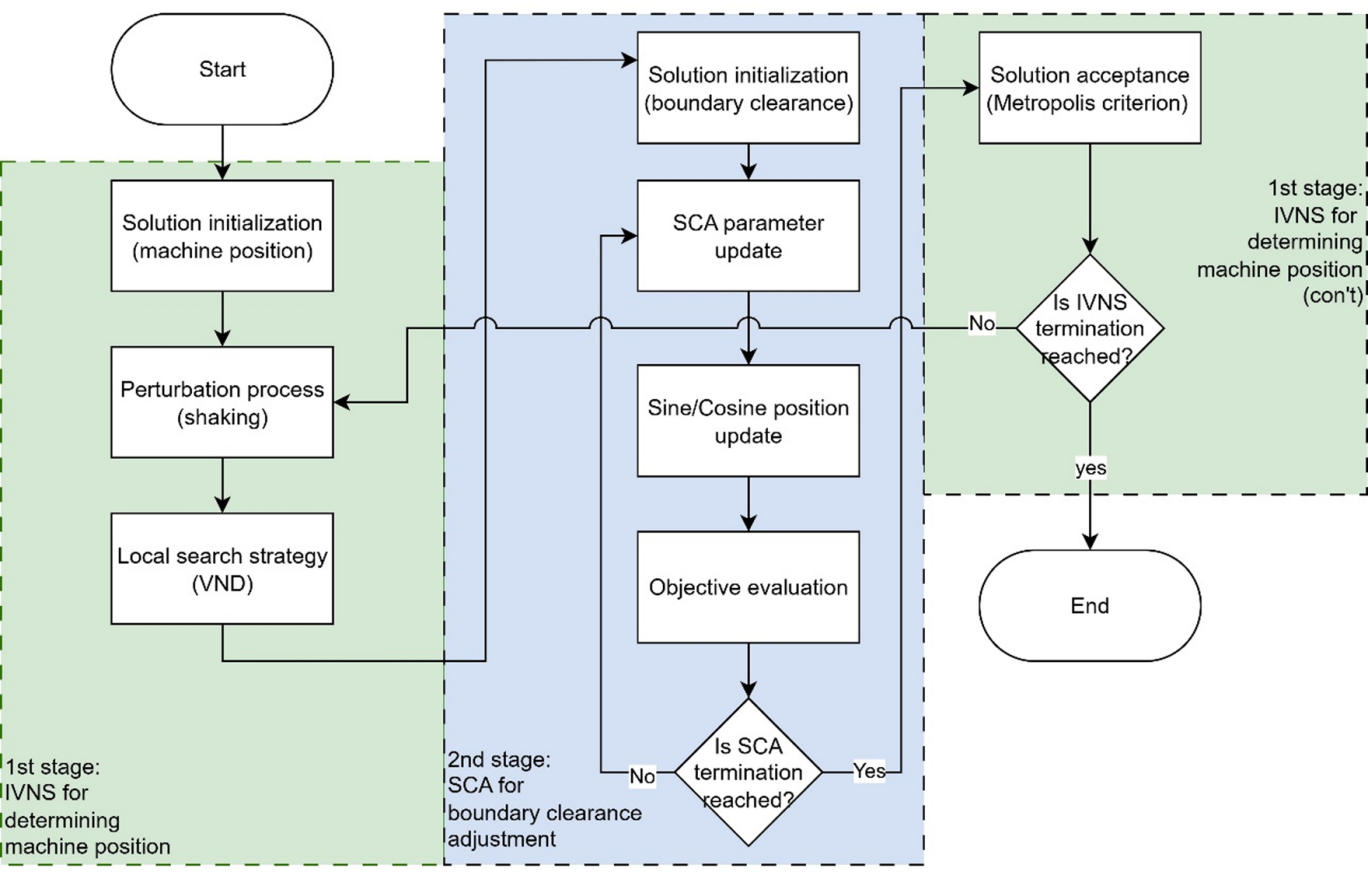
Flowchart of the proposed IVNS-SCA.

The proposed IVNS-SCA consists of two stages: the first stage is IVNS for determining the machine position, while the second stage is SCA for adjusting the boundary clearance and starting point for each row. Figure 2 illustrates the two-stage IVNS–SCA procedure for solving the MRLP with safety considerations. In the first stage (IVNS), the algorithm begins with solution initialization for machine positions, followed by a perturbation process (shaking) and local search using the Variable Neighborhood Descent (VND) strategy. The intermediate solution is then passed to the second stage (SCA), where boundary clearances are initialized, SCA parameters are updated, and starting points are refined through sine/cosine position updates and objective evaluation. This process is iterated until the SCA termination criterion is satisfied. The updated solution is subsequently evaluated using the Metropolis acceptance criterion in IVNS, and the procedure continues until the IVNS termination condition is met, at which point the algorithm ends with the final optimized layout.

### The improved variable neighborhood search

The IVNS is used in the first stage for determining the allocation of machines in each row. Since the concerned MRLP has the characteristic of open number of rows, the algorithm is also used to determine the number of rows in the layout. This algorithm is the extension of variable neighborhood search (VNS) formulated by Mladenovic and Hansen for solving combinatorial optimization problems^35^. The VNS is one of the emerging metaheuristics, and has been applied to various discrete optimization problems, such as minimum linear arrangement problem^49^, and travelling salesman problem with time windows^50^. VNS operates on the principle of iteratively exploring different neighborhoods of solutions within the search space. Unlike traditional algorithms that are confined to a single exploration strategy, VNS dynamically adjusts its search by transitioning between different neighborhood structures. These neighborhoods represent different levels of solution granularity, ranging from local to global configurations. By systematically moving between these neighborhoods, VNS can efficiently navigate complex, multi-dimensional solution spaces, often finding high-quality solutions in a remarkably efficient manner.

The hallmark of VNS lies in its ability to balance exploration and exploitation. During the exploration phase, the algorithm probes diverse regions of the solution space, ensuring that no promising areas are left unexplored. In the exploitation phase, VNS refines its search around the most promising solutions, homing in on optimal or near-optimal solutions with precision. This dynamic interplay between exploration and exploitation enables VNS to escape local optima and discover solutions that are globally optimal or near optimal.

This study adopts the IVNS that first was introduced by Rifai et al.^5^. The IVNS is a variant of skewed VNS (SVNS)^51^, in which an inferior solution can also be permitted as the current solution for next iteration. This procedure is aimed at diversifying the search process to promising regions that are far away from the regions where the search is currently locked^52^. The SVNS has been demonstrated in delivering satisfactory performance in various problems, such as degree constrained minimum spanning tree problem^53^, maximally diverse grouping^54^, cumulative capacitated vehicle routing problem^55^, and location-allocation problem for battery swap station^56^. A recent study by^57^ further improved General Skewed VNS, which demonstrated superior performance when compared to existing leading-edge methods. It set 57 new records for best-known solutions and equaled 43 former records across a collection of 100 instances^57^.

However, one important issue in SVNS is timing of skewed moves whereas premature movement could result in a missed opportunity to explore the region more thoroughly around a current incumbent solution^52^. Here, the improvement was executed by adding the Metropolis criterion for solution acceptance and archive-based solution replacement. These additions enhance the algorithm ability in exploring the search space during early iterations and then deepening the search process in promising regions during late iterations. Further, in this study, the IVNS is improved to accommodate the distinct characteristics of MRLP with open number of rows by tailoring the neighborhood structure and local search strategy. The complete procedure of IVNS for generating the multi-row layout is presented in Algorithm 1.

**Algorithm 1:**
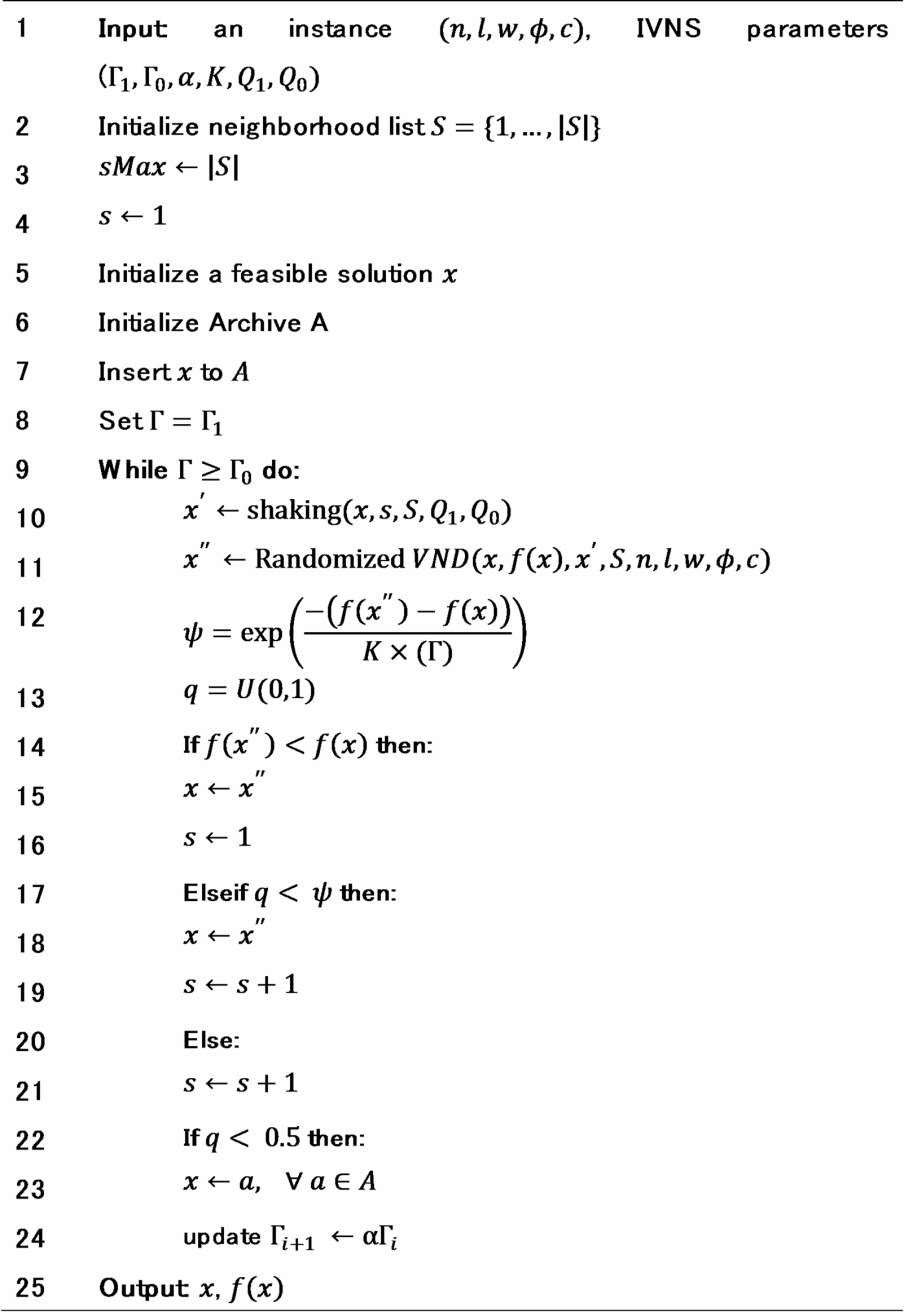
IVNS for the first stage.

The IVNS starts with inputting all required information for generating a layout, which are number of machines $\:n$, length of machine $\:l$, amount of material flow between machines $\:c$, row width $\:w$, and the minimum safe distance between machines $\:\varphi\:$, as listed in Line (1). The aim of this algorithm is to generate a good multi-row layout $\:x$ by minimizing the objective function $\:f\left(x\right)$ which are calculated as the summation between the total material handling cost and the penalty due to violating the minimum safe distance. In addition, the IVNS also requires some parameters, i.e., initial temperature $\:{{\Gamma\:}}_{1}$, final temperature $\:{{\Gamma\:}}_{0}$, cooling rate $\:\alpha\:$, Boltzman constant $\:K$, initial perturbation ratio $\:{Q}_{1}$, and final perturbation ratio $\:{Q}_{0}$.

Lines (2–4) initialize neighborhood list $\:S=\{1,\dots\:,\left|S\right|\}$ which contain $\:sMax$ moves that are deployed in the proposed framework. Here, the first move $\:s=1$ is selected to initialize feasible solution. Afterward, the algorithm generates an initial solution randomly and initialize empty Archive, described in Lines (5–6). Subsequently, the initial solution $\:x$ is stored in archive $\:A$, presented in Line (7). This archive is later used during the acceptance process. The iteration process of IVNS starts with setting the current temperature, as shown in Line (8). In each iteration, the current temperature is updated according to cooling rate $\:{\upalpha\:}$, given in Line (24). The iteration process continues until the termination criterion is satisfied as depicted in Line (9).

The perturbation process of the IVNS in each iteration follows the shaking procedure and local search strategy, described in Lines (10) and (11), respectively. Shaking procedure is performed to generate a modified solution$\:\:{x}^{{\prime\:}}\:$by perturbing the current solution $\:x$ based on the move in $\:N$ neighborhood. The modified solution $\:{x}^{{\prime\:}}$ will then be further exploited by applying local search strategy to generate a new solution$\:\:{x}^{{\prime\:}{\prime\:}}$. Noted that the shaking procedure focuses on the search space exploration process, while local search focuses on the exploitation process, thus balancing between the two.

Subsequently, the new solution is evaluated according to the Metropolis criterion, formulated in Line (12). If the newly generated solution has a better objective than the previous ones, it is automatically accepted as the current solution (Lines (14–16)). However, when the objective of new solution is worse, there are two scenarios: (i) if the value of randomly generated variable $\:q$ is greater than the probability $\:\psi\:$, then the new solution is accepted as the current solution, described in Lines (17–19), (ii) otherwise, the new solution is rejected, and the algorithm decides stochastically to use previous solution or drawn a solution from the archive $\:a\in\:A$ to be set as the current solution, as detailed in Lines (20–23).

An archive mechanism is employed to maintain solution diversity and prevent premature convergence. When a newly generated solution outperforms at least one member of the archive, the worst member (with the highest objective value) is replaced by the new solution. The archive size is fixed at 10, ensuring a balance between preserving high-quality solutions and maintaining diversity to avoid being trapped in local optima. The output of the IVNS is the best-known solution with minimum objective values among all solutions searched, as listed in Line (25).

#### Solution representation

Solution representation holds an important role in metaheuristics since it determines the method for solution modification. To a further extent, the solution representation also affects the efficiency and effectiveness of the method. Therefore, a good solution representation must be simple but can allow extensive mechanisms for solution modification. In this study, a $\:2\times\:n$ matrix is used to represent the multi-row layout solution.

**Fig. 3:**
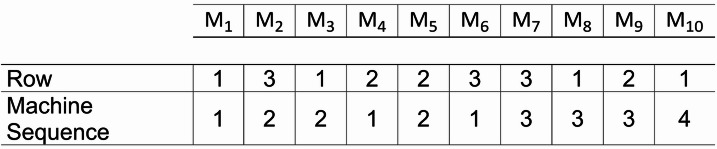
Solution representation for the first stage.

Figure 3 presents an example of solution representation for a 10 machines layout described in Fig. 1. The upper section represents the corresponding row for each machine, i.e., $\:{M}_{2},{M}_{6},{M}_{7}\:$belong to the third row since the three machines have the “Row” values of 3. The lower section represents the position of the machine in the sequence in its assigned row. For example, in third row (Row = 3), $\:{M}_{6}$ is the first machine (Machine Sequence = 1) from the left in that row, followed by $\:{M}_{2}$ (Machine Sequence = 2) and $\:{M}_{7}$ (Machine Sequence = 3). This representation allows inter-row modifications, such as moving the machine to other rows, as well as intra-row modifications, such as modifying the sequence of machines that belong to the same row.

#### Neighborhood structure

**Fig. 4:**
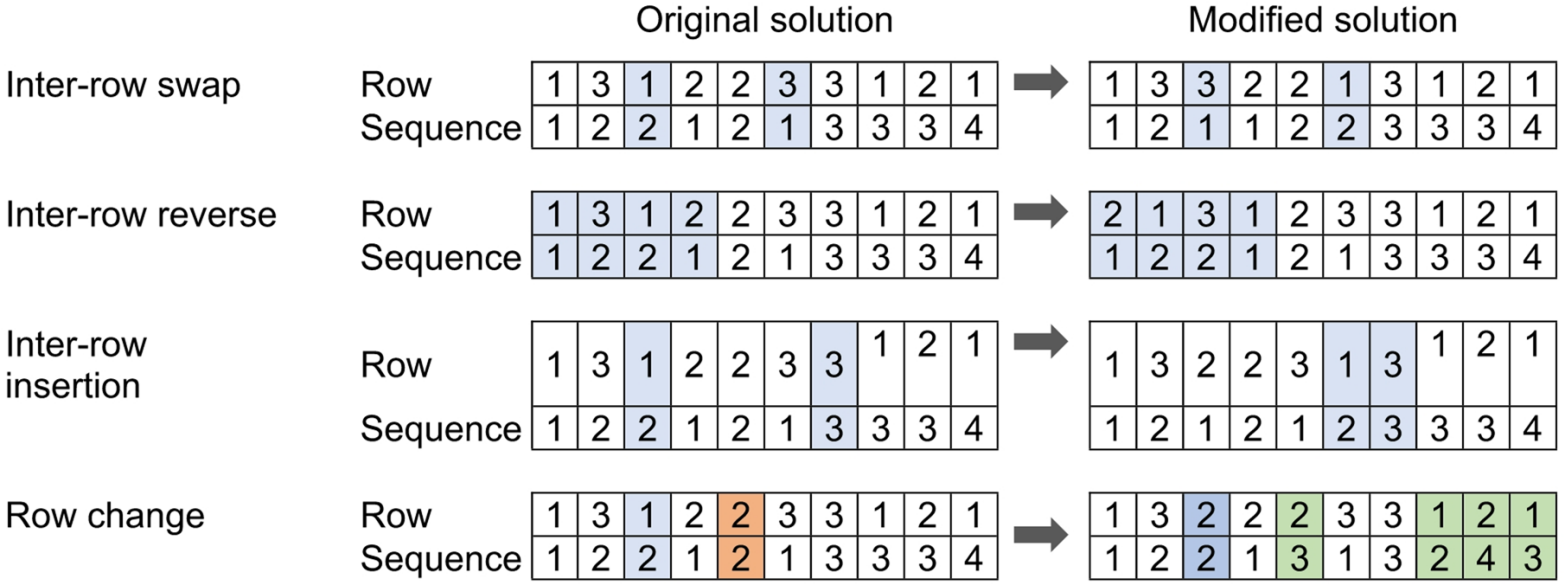
Solution perturbation methods in the proposed IVNS.

The IVNS algorithm is designed to explore various row allocations and machine orders by utilizing four custom neighborhood structures: inter-row swap, inter-row reverse, inter-row insertion, and row change move. These structures are presented in Fig. 4 in the respective order, with nodes subject to change selected randomly, highlighted with the blue blocks. As illustrated in Fig. 4, the change in the inter-row swap and insertion only occurs at selected nodes. However, in the inter-row reverse dan row change moves, the change could also include other nodes. Hence, the inter-row reverse and row change moves have a higher magnitude of perturbation than the inter-row swap and insertion. The decision to execute these moves is based on their magnitudes, to balance the exploration and exploitation of the algorithm in searching for a global optimum solution. These neighborhood structures are used in the shaking procedure as well as in the local search phase of the algorithm.

In addition, the row change moves may generate infeasible solutions. As such, the solution adjustment, highlighted with the green blocks, is performed to ensure the feasibility of the new solutions. The adjustment is performed mainly on sequence value, in which the sequence in a row must follow permutation order. It is done by substituting the redundant value of sequence with unassigned value of sequence. For example, in Fig. 3, the third machine is subject to row change move, highlighted in blue blocks. In the previous solution, machine 3 belongs to the 1st row with sequence number of 2. After row change move, the machine is assigned to the 2nd row with the same sequence number of 2. This creates redundant values since machine 5 also belongs to the 2nd row and sequence number of 2, highlighted in red blocks. As such, the sequence number of machine 5 must be changed to 3, and the sequence number of machine 9 is pushed to 4. Meanwhile, in the 1st row, the sequence number of 2 becomes empty after machine 3 is assigned to another row. Therefore, the sequence number of machines 8 and 10 must be changed from 3 to 4 to 2 and 3, respectively.

#### Local search strategy

The IVNS algorithm uses the Variable Neighborhood Descent (VND) technique to change neighborhoods during its local search phase. As such, the IVNS can be categorized as the improved version of Generalized Variable Neighborhood Descent (GVNS). GVNS is a classic variant of VNS in which one of the VND procedures is selected as the main improvement phase in an iteration, especially during the intensification phase^55^.

**Fig. 5:**
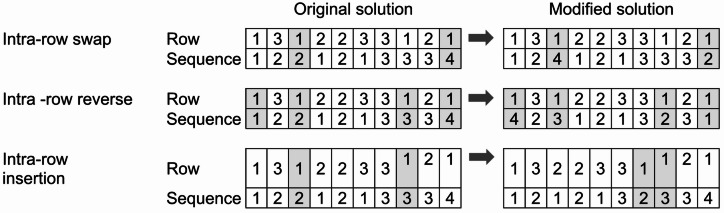
Local search methods in the proposed IVNS.

A VND consists of a set of predefined local search operators that are executed either in a sequential or a nested fashion. Three local search operators are used as exploitation moves: intra-row swap, intra-row reverse, and intra-row insertion moves. These moves refine the machine placement within a row. The process and illustration of the VND operators are shown in Fig. 5. The VND operators are similar to the shaking process moves, but they are performed on machines within the same row, hence emphasizing in intensification during the search process.

In this study, randomized VND is employed to improve the exploitation performance of the algorithm. The main difference between this approach and classical VND is the inclusion of a shuffle procedure in Step 9 of Algorithm 1. When the acceptance criterion is met, the neighborhood list *S* is rearranged. However, in contrast to previous methods, this study randomizes the number of neighborhoods lists *S* at each iteration.

#### Solution acceptance and iteration termination

The Metropolis criterion is adapted as the solution acceptance mechanism in the IVNS. Previously, the Metropolis criterion was embedded for the Simulated Annealing^58^. However, due to its ability in enhancing the exploration of search process, the Metropolis criterion has been widely applied in other metaheuristics as well, such as in Particle Swarm Optimization^59^ and Adaptive Large Neighborhood Search^60^. This mechanism decides whether a newly generated solution$\:\:{x}^{{\prime\:}{\prime\:}}$ is accepted or rejected based on probability which is formulated in the following Eq.

26 $$\:P\left({x}^{{\prime\:}{\prime\:}}\right)=\:\left\{\begin{array}{c}1\:\:\:\:\:\:\:\:\:\:\:\:\:\:\:\:\:\:\:\:\:\:\:\:\:\:\:\:\:\:for\:\:f\left(x{\prime\:}{\prime\:}\right)-f\left(x\right)\:\le\:0\\\:{e}^{-\left(\frac{f\left(x{\prime\:}{\prime\:}\right)-f\left(x\right)}{K\left({{\Gamma\:}}_{p}\right)}\right)}\:\:\:\:\:\:for\:\:f\left(x{\prime\:}{\prime\:}\right)-f\left(x\right)\:>0\end{array}\right.$$

where $\:P\left({x}^{{\prime\:}{\prime\:}}\right)$ is the probability of a new solution $\:{x}^{{\prime\:}{\prime\:}}$ to be accepted. When the new solution has a better objective value than the previous solution, it always has a probability of 1, thus automatically accepted. Meanwhile, when it has a worse objective value, the probability is affected by the deviance against the current objective and the iteration number. Once a new solution is accepted, it will replace the previous solution. Conversely, if the new solution is rejected, there are two options: retain the previous solution or drawing a solution from the Archive to be selected as the current solution. The iteration continues until the current temperature reaches the final temperature.

### The sine cosine algorithm

The second stage aims to adjust the boundary clearance and determines the starting point of each row. Without the second stage, the starting points of machines are determined arbitrarily. Therefore, the algorithm may fail to obtain better solutions. As such, in the proposed two-stage algorithm, the SCA is used to determine the starting points of the rows which return further lower travelled distance.

**Fig. 6:**
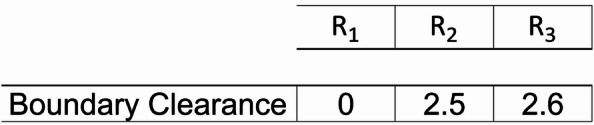
Solution representation for the second stage.

The solution in this stage represents the value of the boundary clearance of each row. Figure 6 presents an example of solution representation for three rows layout described in Fig. 1. Since the first row has the longest length, its boundary clearance is always zero and cannot be modified.

In this study, the Sine Cosine Algorithm (SCA) is used to find the optimal values of the boundary clearance that yield the lowest total material flow cost for the layout that has been generated in the first stage. The SCA is a population-based metaheuristics for continuous problem that is proposed by Mirjalili et al.^61^. It employs two simple solution perturbation processes which, as its name implies, use the two trigonometric functions: sine and cosine functions. The two solution perturbation processes are defined in Eq. 27.

27 $$\:{a}_{j}^{t+1}=\:\left\{\begin{array}{c}{a}_{j}^{t}+{r}_{1}\text{sin}\left({r}_{2}\right)\left|{r}_{3}\left({a}_{j}^{b}\right)-{a}_{j}^{t}\right|\:\:\:\:\:\:\:\:if\:{r}_{4}<0.5\\\:{a}_{j}^{t}+{r}_{1}\text{cos}\left({r}_{2}\right)\left|{r}_{3}\left({a}_{j}^{b}\right)-{a}_{j}^{t}\right|\:\:\:\:\:\:\:\:if\:{r}_{4}\ge\:0.5\end{array}\right.$$

Where $\:{a}_{j}^{t}$ is the current solution (the boundary clearance) for row $\:j\in\:R$ at iteration $\:t$, $\:{a}_{j}^{b}$ is the best-known solution so far for those rows, and $\:{a}_{j}^{t+1}$ is the modified solution which can be generated by the two processes. Noted that in each iteration for each row, only one perturbation process can be used, which is randomly selected by generating random number $\:{r}_{4}=U\left[\text{0,1}\right]$. As such, both perturbation processes have equal probabilities to be selected.

28 $$\:{r}_{1}=\alpha\:-t\left(\frac{\alpha\:}{T}\right)$$

29 $$\:{r}_{2}=U\left[\text{0,2}\pi\:\right]$$

30 $$\:{r}_{3}=U\left[\text{0,2}\right]$$

Besides the trigonometric function, the perturbation process is also affected by the three SCA parameters: $\:{r}_{1},{r}_{2}$ and $\:{r}_{3}$, calculated in Eqs. (28–30), respectively. Parameter $\:{r}_{1}$ balances exploration and exploitation during the search process. Its value is governed by the pre-determined constant parameter $\:\alpha\:$, the number of current iterations $\:t$, and the total iterations $\:T$. Parameter $\:{r}_{2}$ directs the movement of search process. Parameter $\:{r}_{3}$ stochastically emphasizes or deemphasizes the effect of $\:{a}_{j}^{b}$ during the perturbation. The detailed process of SCA for boundary clearance determination is explained in Algorithm 2.

**Algorithm 2:**
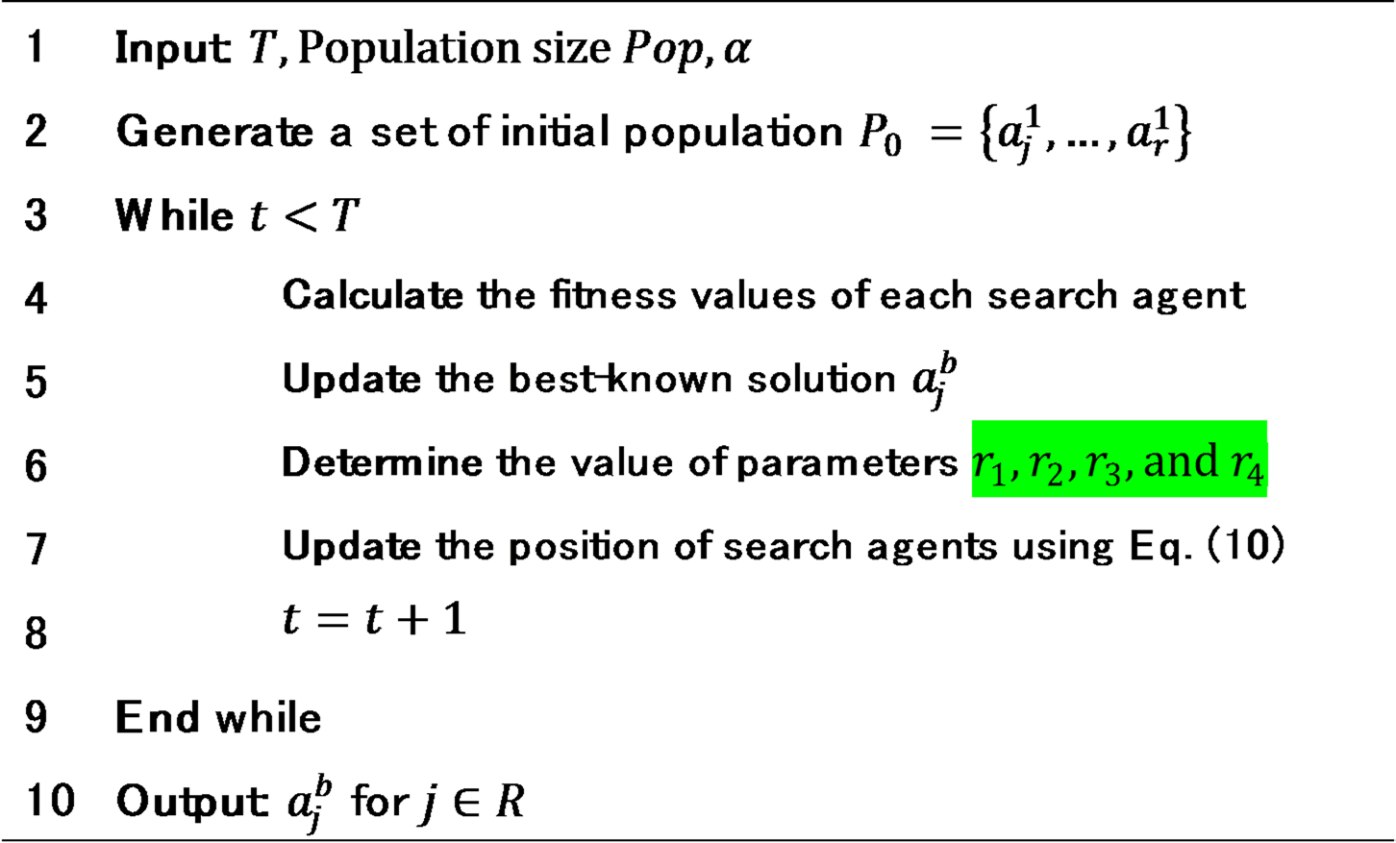
SCA for the second stage.

## Experiment and result

This section discusses the experiment to evaluate the performance of the proposed method. The evaluation of the method is executed in experiment with several instances. The algorithms are developed in MATLAB R2021a on a personal computer with Intel^®^ CoreTM i5-10400 F CPU @2.90 GHz (12 CPUs) and 16GB of RAM. The computation is only executed on a single core of the computer.

### Test problems and parameter setting

The dataset for the test is taken from Rifai et al.^6^, consisting of 34 instances with various complexity with $\:n\in\:\left\{\text{11,12,13,14,15,17,40}\right\}.$ The recommended safe distances between machines are randomly generated according to the discrete uniform distribution for half of the pair relationship. As for the other half, the recommended safe distance is zero $\:{\varphi\:}_{ji}=0$, which means that the two machines can be placed in proximity. The matrix is symmetrical, in which $\:{\varphi\:}_{ij}={\varphi\:}_{ji}$.

To ensure fairness during the experiment, all algorithms are executed in the same condition. In addition, the algorithms are executed under comparable computation time, following the computation time of the proposed IVNS. As such, the iteration process for SA and GVNS will be terminated once the current time reaches the computation time of the IVNS, albeit it has not reached the final temperature and maximum iteration. Table 2 details the parameter values of the methods.

**Table 2:** The parameter values of the methods.

Parameter	Meaning	Value
$\:{{\Gamma\:}}_{1}$	Initial temperature	1
$\:{{\Gamma\:}}_{0}$	Final temperature	0.01
$\:{I}_{n}$	Maximum no improvement iteration	20,000
$\:\alpha\:$	Cooling rate	0.995
$\:{I}_{\text{m}\text{a}\text{x}}$	Maximum iteration at a temperature	200
$\:K$	Boltzmann constant	1/40
$\:{Q}_{1}$	Initial perturbation ratio	0.5
$\:{Q}_{0}$	Final perturbation ratio	0.05
$\:T$	Number of maximum generations	500
$\:a$	Constant parameter	2
$\:b$	Constant parameter	2
$\:Pop$	Population size	50

### Evaluation metrics

The performances of the proposed and benchmark methods are evaluated using the Relative Deviation Index (RDI) metric, given as follows.

31 $$\:{RDI}_{A}=\frac{{f}_{A}-{f}_{best}}{{f}_{worst}-{f}_{best}}\times\:100$$

where $\:{RDI}_{A}$ is the Relative Deviation Index of algorithm A. $\:{f}_{A}$ is the objective obtained by algorithm A, while $\:{f}_{best}$ and $\:{f}_{worst}$ are the best (minimum) and the worst (maximum) objectives obtained by all methods in that instance, respectively. The RDI value ranges between 0 and 100, in which lower value of RDI indicates a good solution with better objective than the higher RDI value.

In addition, the use of SCA automatically adds the computation time, thus it is important to assess its worthiness to be embedded in the framework. Here, the average percentage deviation (API) is used to calculate the improvement obtained by the two-stage method against the standard method without adding SCA for the second stage. Equation (32) presents the formulation of API. The deviation is assessed by the RDI value.

32 $$\:{API}_{A-B}=\frac{{RDI}_{A}-{RDI}_{B}}{{RDI}_{B}}\times\:100$$

where $\:{RDI}_{A}$ is the Relative Deviation Index of the two-stage algorithm (i.e. IVNS-SCA), while $\:{RDI}_{B}$ is the Relative Deviation Index of the standard method using the no adjustment rule for the second stage (i.e. IVNS only). Besides the RDI, the API is also used to assess the increase of computation time by introducing the SCA in the framework by simply replacing $\:{RDI}_{A}$ with the average computation time of two-stage algorithm and replacing $\:{RDI}_{B}$ with the standard algorithm in Eq. (31).

### Implication of considering safe distance

**Fig. 7:**
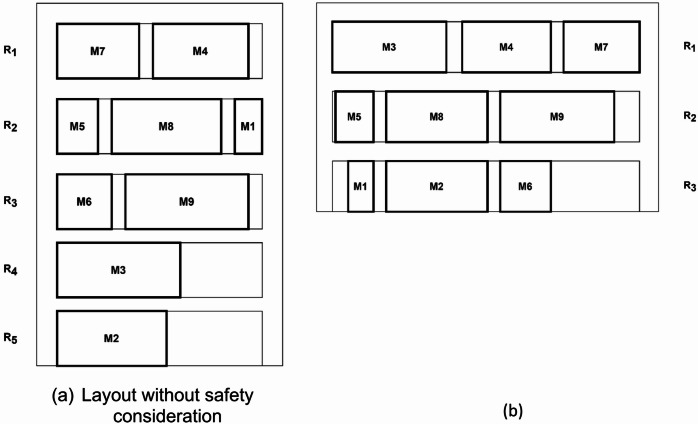
(**a**) The proposed layouts for instance 1 ($\:n=9)$. (**b**) Layout with safety consideration.

This subsection presents the importance of developing a model that can consider the inclusion of safe distance in the MRLP. The addition of recommended safe distance adds complexity to the problem. As presented in Sect. 3, the safe distance is accommodated in the second objective which imposes penalty if the safe distance is violated. In the standard MRLP, the model simply aims to minimize the material handling cost, disregarding the violation of safe distance. In contrast, the proposed model aims to minimize both the material handling cost and the penalty due to safe distance violation. Here, two results of multi-row layout for instance 1 ($\:n=9)$, with the standard multi-row model and the multi-row model with safety consideration generated by the IVNS-SCA are illustrated in Fig. 7. Table 3 presents the recommended safe distance between machines in instance 1, with the row width $\:w=3$.

**Table 3:** The recommended minimum safe distance $\:\varphi\:$ in instance 1.

	$\:{M}_{1}$	$\:{M}_{2}$	$\:{M}_{3}$	$\:{M}_{4}$	$\:{M}_{5}$	$\:{M}_{6}$	$\:{M}_{7}$	$\:{M}_{8}$	$\:{M}_{9}$
$\:{M}_{1}$	–	0	11	0	0	12	13	13	16
$\:{M}_{2}$	0	–	18	0	0	20	20	19	19
$\:{M}_{3}$	11	18	–	0	0	19	0	12	15
$\:{M}_{4}$	0	0	0	–	13	13	0	20	0
$\:{M}_{5}$	0	0	0	13	–	15	0	0	12
$\:{M}_{6}$	12	20	19	13	15	–	14	19	0
$\:{M}_{7}$	13	20	0	0	0	14	–	15	0
$\:{M}_{8}$	13	19	12	20	0	19	15	–	0
$\:{M}_{9}$	16	19	15	0	12	0	0	0	–

Figure 7 presents two different layouts, for instance 1: a layout without safety consideration (Fig. 7a) and a layout without safety consideration (Fig. 7b). Layout (a) has five rows, with relatively short row length, while Layout (b) has three rows with longer row length. In the perspective of material handling cost, Layout (a) gives better cost at $\:{f}_{1}=\text{1,959.5}$, while Layout (b) has $\:{f}_{1}=\text{2,865}.$ However, Layout (a) violates the safe distance. For example, $\:{M}_{1}$ and $\:{M}_{8}$ are placed side by side in Layout (a) while it should not be according to the recommended minimum safe distance in Table 4. In another case, the placement of $\:{M}_{2}$ and $\:{M}_{3}$ is also violates the safe distance in which they are placed adjacent to each other. Meanwhile, albeit having higher material handling cost, Layout (b) can accommodate this issue by placing $\:{M}_{2}$ and $\:{M}_{3}$ to be far apart from each other, in $\:{R}_{3}$ and $\:{R}_{1}$ for $\:{M}_{2}$ and $\:{M}_{3}$, respectively. This illustration highlights that it is important to develop a model that can inclusively consider the issue of safe distance.

### Algorithms evaluation

#### Effectiveness of IVNS-SCA

The proposed algorithm is compared against three benchmarks methods for the first stage: Gurobi solver, SA adopted from Kirkpatrick et al.^56^ and conventional GVNS adopted from Karakostas and Sifaleras^62^ with modifications in the perturbation operators to suit the MRLP. The optimization using Gurobi was performed with a time limit of 14,400 s, MIPGap tolerance of $\:1\times\:{10}^{-4}$, and single-threaded computation to ensure comparability with the metaheuristic algorithms, which were also run on a single CPU core. The average objective value and the percentage improvement obtained by IVNS-SCA against benchmark metaheuristics (SA, SA-SCA, GVNS, GVNS-SCA, IVNS) are presented in Tables 4 and 5. In addition, the average computation time of the methods in each instance is presented in Table 6.

**Table 4:** The average objective values of all methods.

Instance	Gurobi solver		Metaheuristics						
	Objective	Optimality gap (%)	SA	SA-SCA	GVNS	GVNS-SCA	IVNS	IVNS-SCA	
1	6,693	77.70%	2,921	2,914	2,921	2,904	2,850	2,836	
2	7,652	80.62%	3,883	3,873	3,977	3,954	3,897	3,880	
3	8,265	92.05%	3,745	3,729	3,738	3,715	3,782	3,747	
4	–	–	7,676	7,583	7,573	7,485	7,392	7,295	
5	–	–	6,087	6,043	6,161	6,125	6,041	6,003	
6	–	–	7,131	7,115	7,163	7,117	7,106	7,053	
7	–	–	7,728	7,679	7,857	7,781	7,763	7,684	
8	–	–	4,800	4,797	4,963	4,954	4,793	4,780	
9	–	–	4,127	4,115	4,192	4,175	4,105	4,099	
10	–	–	5,472	5,441	5,855	5,840	5,498	5,473	
11	–	–	5,491	5,476	5,586	5,564	5,424	5,386	
12	–	–	4,443	4,436	4,497	4,490	4,292	4,282	
13	–	–	8,872	8,855	9,269	9,254	8,818	8,798	
14	–	–	8,510	8,490	8,767	8,758	8,503	8,476	
15	–	–	7,662	7,638	7,997	7,988	7,705	7,690	
16	–	–	8,473	8,401	8,551	8,530	8,399	8,352	
17	–	–	9,620	9,535	9,780	9,745	9,521	9,497	
18	–	–	10,273	10,224	10,303	10,275	10,188	10,163	
19	–	–	10,824	10,758	11,365	11,315	10,864	10,784	
20	–	–	12,876	12,789	12,822	12,759	12,994	12,871	
21	–	–	10,603	10,560	10,843	10,796	10,611	10,508	
22	–	–	8,909	8,846	9,624	9,506	8,958	8,913	
23	–	–	9,184	9,154	9,402	9,365	9,053	9,030	
24	–	–	5,952	5,890	6,244	6,109	6,008	5,898	
25	–	–	12,362	12,293	12,630	12,566	12,168	12,112	
26	–	–	16,001	15,910	16,674	16,557	15,984	15,819	
27	–	–	16,588	16,558	17,681	17,500	16,810	16,660	
28	–	–	120,535	119,166	136,597	132,527	119,035	117,602	
29	–	–	151,374	148,047	169,289	162,967	146,950	144,194	
30	–	–	360,905	352,182	371,817	361,785	359,437	351,346	
31	–	–	203,846	201,084	225,134	215,143	203,806	199,768	
32	–	–	246,067	242,161	266,110	259,119	244,814	241,106	
33	–	–	5,467,281	4,997,185	5,992,804	5,273,311	5,094,405	4,772,603	
34	–	–	1,267,351	1,177,087	1,267,045	1,109,376	1,209,712	1,129,118	

**Table 5:** Percentage improvement of IVNS-SCA against benchmark metaheuristics.

Instance	Improvement of IVNS-SCA against benchmark metaheuristics				
	vs. SA	vs. SA-SCA	vs. GVNS	vs. GVNS-SCA	vs. IVNS
1	2.9%	2.7%	2.9%	2.3%	0.5%
2	0.1%	− 0.2%	2.4%	1.9%	0.4%
3	− 0.1%	− 0.5%	− 0.2%	− 0.9%	0.9%
4	5.0%	3.8%	3.7%	2.5%	1.3%
5	1.4%	0.7%	2.6%	2.0%	0.6%
6	1.1%	0.9%	1.5%	0.9%	0.7%
7	0.6%	− 0.1%	2.2%	1.2%	1.0%
8	0.4%	0.4%	3.7%	3.5%	0.3%
9	0.7%	0.4%	2.2%	1.8%	0.1%
10	0.0%	− 0.6%	6.5%	6.3%	0.5%
11	1.9%	1.6%	3.6%	3.2%	0.7%
12	3.6%	3.5%	4.8%	4.6%	0.2%
13	0.8%	0.6%	5.1%	4.9%	0.2%
14	0.4%	0.2%	3.3%	3.2%	0.3%
15	− 0.4%	− 0.7%	3.8%	3.7%	0.2%
16	1.4%	0.6%	2.3%	2.1%	0.6%
17	1.3%	0.4%	2.9%	2.5%	0.3%
18	1.1%	0.6%	1.4%	1.1%	0.2%
19	0.4%	− 0.2%	5.1%	4.7%	0.7%
20	0.0%	− 0.6%	− 0.4%	− 0.9%	0.9%
21	0.9%	0.5%	3.1%	2.7%	1.0%
22	0.0%	− 0.8%	7.4%	6.2%	0.5%
23	1.7%	1.4%	4.0%	3.6%	0.3%
24	0.9%	− 0.1%	5.5%	3.5%	1.8%
25	2.0%	1.5%	4.1%	3.6%	0.5%
26	1.1%	0.6%	5.1%	4.5%	1.0%
27	− 0.4%	− 0.6%	5.8%	4.8%	0.9%
28	2.4%	1.3%	13.9%	11.3%	1.2%
29	4.7%	2.6%	14.8%	11.5%	1.9%
30	2.6%	0.2%	5.5%	2.9%	2.3%
31	2.0%	0.7%	11.3%	7.1%	2.0%
32	2.0%	0.4%	9.4%	7.0%	1.5%
33	12.7%	4.5%	20.4%	9.5%	6.3%
34	10.9%	4.1%	10.9%	− 1.8%	6.7%
Average	1.9%	0.9%	5.3%	3.7%	1.1%

**Table 6:** The computation times of all methods.

Instance	Average computation time (s)					
	SA	SA-SCA	GVNS	GVNS -SCA	IVNS	IVNS-SCA
1	64.5	65.0	64.52	65.01	64.52	65.02
2	64.4	64.9	64.44	64.93	64.44	64.94
3	70.3	70.8	70.27	70.83	70.27	70.84
4	78.3	79.0	78.33	79.00	78.32	78.99
5	82.5	83.1	82.47	83.15	82.47	83.15
6	75.6	76.2	75.58	76.25	75.58	76.24
7	76.6	77.3	76.64	77.28	76.64	77.28
8	70.3	71.0	70.34	70.96	70.34	70.95
9	341.8	342.5	341.85	342.53	341.85	342.56
10	84.4	85.1	84.37	85.07	84.37	85.07
11	75.3	76.0	75.29	76.00	75.29	76.00
12	86.1	86.9	86.06	86.86	86.06	86.83
13	82.7	83.4	82.69	83.42	82.69	83.43
14	82.4	83.2	82.43	83.17	82.43	83.18
15	82.8	83.5	82.77	83.53	82.77	83.53
16	78.7	79.5	78.74	79.49	78.74	79.48
17	84.8	85.6	84.81	85.65	84.81	85.64
18	89.5	90.3	89.46	90.29	89.46	90.31
19	90.2	91.1	90.20	91.05	90.20	91.05
20	92.4	93.2	92.36	93.22	92.36	93.23
21	105.3	106.2	105.26	106.24	105.26	106.24
22	106.1	107.0	106.09	107.05	106.09	107.05
23	102.6	103.6	102.61	103.64	102.61	103.64
24	102.2	103.2	102.19	103.19	102.19	103.18
25	103.0	104.0	102.97	104.06	102.97	104.04
26	121.0	122.3	121.01	122.33	121.01	122.34
27	126.8	128.2	126.78	128.25	126.78	128.22
28	405.9	410.2	434.78	439.08	405.92	410.21
29	317.2	321.6	340.01	344.34	317.23	321.60
30	303.7	307.9	339.64	343.82	303.72	307.89
31	315.5	319.8	349.30	353.66	315.52	319.85
32	324.7	329.1	350.41	354.71	324.70	329.05
33	323.2	327.6	353.98	358.36	323.24	327.61
34	313.5	317.8	346.12	350.41	313.49	317.74

Table 4 indicates that the solutions obtained by Gurobi were significantly suboptimal, even for the simplest instances, with recorded optimality gaps of 77.70%, 80.62%, and 92.05% for instances 1 through 3, respectively. In contrast, the proposed metaheuristic consistently outperformed the Gurobi solver, even on the simplest instance. These results underscore the problem’s complexity and highlight the critical need for developing effective metaheuristic approaches to address it.

Table 5 shows that IVNS–SCA achieves the most notable improvements when compared to GVNS-based methods, averaging 5.3% against GVNS and 3.7% against GVNS–SCA. This trend is consistent across many instances, with particularly large gains in complex cases such as instance 28 (+ 13.9%), instance 29 (+ 14.8%), and instance 33 (+ 20.4%). In contrast, the improvements over SA and SA–SCA are much smaller, averaging 1.9% and 0.9%, with some instances even showing slight negative values (e.g., instances 3, 15, 20). Against IVNS without adjustment, the improvement is modest at 1.1%, indicating that the added SCA stage brings incremental rather than dramatic benefits over its base version.

Another clear pattern is the influence of problem size. In large-scale instances (28–34), IVNS–SCA delivers substantial gains, often exceeding 10%, while in smaller instances, the improvements are generally below 3% and sometimes negative. This suggests that SCA’s contribution is most effective when the number of machines and rows increases, giving more opportunity for optimizing boundary clearance. Overall, the table highlights that IVNS–SCA is consistently stronger than GVNS-based methods and provides meaningful benefits in larger, more complex cases.

Table 6 shows that the computation times of SA, GVNS, and IVNS are almost identical to their hybrid counterparts (SA–SCA, GVNS–SCA, and IVNS–SCA). For most instances, the addition of SCA increases runtime by less than one second (e.g., instance 1: 64.5s vs. 65.0s, instance 12: 86.1s vs. 86.9s). This pattern is consistent across all cases, demonstrating that integrating SCA as a fine-tuning stage has only a minimal effect on total computation time.

In larger instances (28–34), the overall runtime naturally rises (300–400 s), but the difference between single-stage and two-stage methods remains very small—typically within 1–2%. For example, in instance 28, IVNS takes 405.9s while IVNS–SCA requires 410.2s. This confirms that the benefits of SCA in improving solution quality come at a negligible computational cost, making the two-stage approach practical even for large and complex problem sizes.

#### Impact of SCA

For all metaheuristics computations, the performance of SCA is compared against the scenario without the adjustment of boundary clearance in the second stage. In the no adjustment scenario, the starting points of machines are determined arbitrarily. Hence, there are six algorithms to be evaluated: SA-no adjustment, SA-SCA, GVNS-no adjustment, GVNS -SCA, IVNS-no adjustment, and IVNS-SCA.

The comparison is executed to prove two aims: the significance of including SCA for the second stage, and the improvement of proposed IVNS-SCA against the benchmark methods. For each instance, ten replications are executed. Table 7 summarizes the average RDI of the methods in each instance. The best value for each instance is highlighted in bold font.

**Table 7:** RDI of all methods in all instances.

Instance	$\:n$	SA		GVNS		IVNS	
		No adjustment	SCA	No adjustment	SCA	No adjustment	SCA
1	9	56.57	55.09	56.55	52.94	40.75	**37.56**
2	9	30.23	**28.53**	46.56	42.57	32.76	29.84
3	10	57.41	55.63	56.66	**54.11**	61.53	57.62
4	11	44.39	40.31	39.88	36.08	32.00	**27.77**
5	11	32.67	28.36	39.92	36.39	28.15	**24.48**
6	11	34.13	32.73	36.92	32.86	31.88	**27.28**
7	11	41.64	**38.17**	50.81	45.40	44.16	38.48
8	11	18.86	18.34	48.06	46.40	17.69	**15.34**
9	11	32.55	30.34	44.30	41.17	28.59	**27.42**
10	11	19.81	**16.79**	56.76	55.30	22.25	19.87
11	12	42.01	39.51	57.82	54.19	30.78	**24.56**
12	12	52.51	51.50	59.70	58.80	32.15	**30.81**
13	12	31.37	29.79	67.78	66.45	26.46	**24.56**
14	12	26.94	24.86	53.35	52.36	26.26	**23.47**
15	12	13.13	**10.49**	50.01	49.08	17.88	16.28
16	12	37.60	30.17	45.80	43.60	29.95	**25.05**
17	13	34.95	27.99	47.93	45.03	26.90	**24.90**
18	13	56.25	52.84	58.40	56.44	50.36	**48.54**
19	13	16.63	**12.51**	50.20	47.11	19.11	14.14
20	13	45.32	40.35	42.22	**38.63**	52.14	45.08
21	13	44.73	40.21	70.30	65.31	45.59	**34.62**
22	13	27.30	**24.49**	58.89	53.67	29.44	27.47
23	14	35.71	33.13	54.27	51.16	24.53	**22.55**
24	14	42.85	**39.34**	59.48	51.79	46.08	39.77
25	15	30.40	26.41	45.95	42.21	19.18	**15.94**
26	17	29.62	26.41	53.05	48.98	29.01	**23.25**
27	18	24.83	**23.85**	60.44	54.53	32.07	27.17
28	40	18.98	15.17	63.71	52.38	14.81	**10.81**
29	40	26.43	21.06	55.32	45.13	19.30	**14.85**
30	40	42.41	32.51	54.80	43.41	40.74	**31.56**
31	40	22.51	18.17	55.99	40.28	22.45	**16.10**
32	40	25.72	18.05	65.12	51.38	23.26	**15.97**
33	40	46.54	35.45	58.94	41.96	37.74	**30.15**
34	40	33.92	23.74	33.88	**16.11**	27.42	18.34
Average		34.62	30.66	52.94	47.45	31.28	**26.81**

The SA, GVNS, and IVNS are the methods in the first stage to determine the number of rows and the machine sequence in each row. The SCA and ‘No adjustment’ are the method for the second stage to determine the starting point of each row. Noted that the ‘No adjustment’ means that there is no adjustment on the boundary clearance, hence starting points of all rows are at point zero in *x*-axis. The result indicates that the proposed two-stage IVNS-SCA clearly outperforms the other algorithms, depicted by the lowest average RDI. Among 34 instances, the IVNS-SCA can obtain the best results in 23 instances.

Considering the equal computation time between the three methods, the result shows the ability of the proposed IVNS-SCA in finding better solutions. The result also clearly shows the improvement obtained by the IVNS against the conventional GVNS, albeit using the same neighborhood structure and local search strategy. It indicates that the introduction of Metropolis criterion into the IVNS framework is significantly useful to enhance the exploration ability of the algorithm, hence preventing premature convergence.

**Table 8:** The API in computation time and RDI.

Instance	API of computation time			API of RDI		
	SA-SCA vs. SA	GVNS-SCA vs. GVNS	IVNS-SCA vs. IVNS	SA-SCA vs. SA	GVNS -SCA vs. GVNS	IVNS-SCA vs. IVNS
1	0.76%	0.76%	0.78%	− 2.62%	− 6.38%	− 7.82%
2	0.77%	0.76%	0.78%	− 5.61%	− 8.57%	− 8.92%
3	0.81%	0.80%	0.81%	− 3.09%	− 4.50%	− 6.36%
4	0.86%	0.86%	0.85%	− 9.20%	− 9.54%	− 13.21%
5	0.83%	0.83%	0.83%	− 13.20%	− 8.84%	− 13.05%
6	0.83%	0.88%	0.87%	− 4.10%	− 11.01%	− 14.44%
7	0.85%	0.84%	0.85%	− 8.33%	− 10.64%	− 12.87%
8	0.88%	0.88%	0.87%	− 2.77%	− 3.44%	− 13.33%
9	0.20%	0.20%	0.21%	− 6.78%	− 7.06%	− 4.09%
10	0.84%	0.83%	0.84%	− 15.26%	− 2.56%	− 10.72%
11	0.95%	0.95%	0.94%	− 5.96%	− 6.29%	− 20.23%
12	0.94%	0.92%	0.89%	− 1.93%	− 1.51%	− 4.15%
13	0.89%	0.88%	0.89%	− 5.03%	− 1.96%	− 7.18%
14	0.89%	0.90%	0.91%	− 7.73%	− 1.86%	− 10.62%
15	0.91%	0.91%	0.91%	− 20.12%	− 1.86%	− 8.96%
16	0.95%	0.95%	0.94%	− 19.74%	− 4.80%	− 16.37%
17	0.97%	0.99%	0.98%	− 19.90%	− 6.03%	− 7.42%
18	0.93%	0.93%	0.95%	− 6.07%	− 3.36%	− 3.60%
19	0.95%	0.94%	0.94%	− 24.78%	− 6.16%	− 25.98%
20	0.93%	0.92%	0.94%	− 10.98%	− 8.52%	− 13.54%
21	0.90%	0.93%	0.93%	− 10.10%	− 7.11%	− 24.06%
22	0.90%	0.91%	0.90%	− 10.29%	− 8.87%	− 6.70%
23	1.00%	1.01%	1.00%	− 7.23%	− 5.72%	− 8.04%
24	0.99%	0.98%	0.98%	− 8.18%	− 12.93%	− 13.70%
25	1.05%	1.06%	1.04%	− 13.15%	− 8.12%	− 16.90%
26	1.09%	1.09%	1.10%	− 10.81%	− 7.68%	− 19.84%
27	1.14%	1.16%	1.14%	− 3.94%	− 9.78%	− 15.27%
28	1.06%	0.99%	1.06%	− 20.08%	− 17.79%	− 26.96%
29	1.37%	1.27%	1.38%	− 20.30%	− 18.43%	− 23.03%
30	1.39%	1.23%	1.38%	− 23.35%	− 20.79%	− 22.55%
31	1.37%	1.25%	1.37%	− 19.30%	− 28.06%	− 28.29%
32	1.35%	1.23%	1.34%	− 29.85%	− 21.10%	− 31.33%
33	1.35%	1.24%	1.35%	− 23.83%	− 28.80%	− 20.12%
34	1.38%	1.24%	1.36%	− 30.00%	− 52.46%	− 33.13%
Average	0.98%	0.96%	0.98%	− 11.44%	− 10.37%	− 14.27%

Further, the effectiveness of using SCA for the second stage is also evaluated. Table 8 presents the average percentage deviation API for the computation time and the obtained RDI between the two-stage methods (SA-SCA, GVNS-SCA, and IVNS-SCA) and their standard methods (with no adjustment) counterparts. The results clearly indicate that the use of SCA for the second stage can significantly reduce the RDI with an average of 11.44%, 10.37%, and 14.27% for the SA, GVNS, and IVNS, respectively. The improvement is especially prominent in large-size instances since there are a greater number of rows that its starting points need to be adjusted. However, this improvement is compensated for by the increase of computation time with average of 0.98%, 0.96%, 0.98% for SA-SCA, GVNS-SCA, and IVNS-SCA against their standard method counterparts, respectively. Considering that the increase of computation time by adding the SCA is generally low (below 1.5%) while enabling significant improvement of RDI, the two-stage methods are deemed to be more favorable to be applied for solving the MRLP with safe distance consideration.

### Statistical analysis

Statistical analysis is performed to further evaluate the advantage of the proposed IVNS–SCA as compared to the benchmark algorithms in terms of the obtained RDI values. The simple two-tailed t-tests are performed for each comparison using the hypothesis as follows.

33 $$\:{H}_{0}:\:{\mu\:}_{A}^{RDI}=\:{\mu\:}_{B}^{RDI}$$

34 $$\:{H}_{1}:\:{\mu\:}_{A}^{RDI}\ne\:{\mu\:}_{B}^{RDI}$$

where $\:{\mu\:}_{A}^{RDI}$ is the mean of RDI obtained by the IVNS – SCA, while $\:{\mu\:}_{B}^{RDI}$ is the mean of RDI obtained by the benchmark algorithm, for $\:B=\{\text{I}\text{V}\text{N}\text{S},\:\text{S}\text{A},\:\text{S}\text{A}-\text{S}\text{C}\text{A},\:\text{G}\text{V}\text{N}\text{S},\:\text{G}\text{V}\text{N}\text{S}-\text{S}\text{C}\text{A}\}$. The alpha is 0.05 which means that if the *p-*value is less than 0.05, the $\:{H}_{0}$ is rejected and $\:{H}_{1}$ is accepted. It means that there is a significant difference between the Means of the two algorithms RDI. Table 9 presents the results of the statical tests.

**Table 9:** The results of the statistical tests.

Algorithm 1	Algorithm 2	*p*-value	Description
IVNS – SCA	SA – No adjustment	0.0053	$\:{H}_{0}$ is rejected; the IVNS-SCA is significantly better
IVNS – SCA	SA – SCA	0.1612	Fail to reject $\:{H}_{0}$
IVNS – SCA	GVNS – No adjustment	$\:1.03\times\:{10}^{-16}$	$\:{H}_{0}$ is rejected; the IVNS-SCA is significantly better
IVNS – SCA	GVNS – SCA	$\:4.06\times\:{10}^{-12}$	$\:{H}_{0}$ is rejected; the IVNS-SCA is significantly better
IVNS – SCA	IVNS – No adjustment	0.0913	Fail to reject $\:{H}_{0}$

The tests indicate that the IVNS – SCA outperforms three benchmark algorithms: SA – No adjustment, GVNS – No adjustment, and GVNS – SCA. Especially against the GVNS based algorithm, this result confirms the significance of improvement obtained by the proposed IVNS. Subsequently, the test fails to reject $\:{H}_{0}$ in the comparison against two benchmark algorithms: SA – SCA, IVNS – No adjustment which means that the improvement is not as significant as against the other algorithms. Specifically, in smaller or less complex instances, the adjustment of boundary clearance by SCA has limited effect, since the row lengths and machine placements leave fewer opportunities for meaningful improvement. Similarly, when the initial layouts generated by the first-stage algorithm (e.g., IVNS) are already near-optimal, the contribution of SCA becomes marginal, leading to non-significant differences in Table 9.

On the other hand, the advantage of SCA becomes more apparent in large-scale instances with many rows or more diverse machine sizes, where boundary clearance decisions have greater influence on total material handling cost. In these cases, SCA significantly enhances layout quality, as reflected in the substantial Relative Deviation Index (RDI) reduction observed in large instances (Tables 7 and 8). Nevertheless, considering that all algorithms run on similar computation time, the IVNS – SCA performs favorably by obtaining the best objective value in most of the instances.

### Discussion

The MRLP is inherently complex due to the simultaneous need to optimize machine sequencing within rows, inter-row arrangements, and safety clearances. Exact methods quickly become intractable as the number of machines increases, which motivates the use of advanced metaheuristics. Based on numerical experiments on 34 benchmark instances, the proposed IVNS–SCA consistently achieved higher-quality solutions compared to SA, GVNS, and their hybrid versions. These results demonstrate that combining IVNS for machine sequencing with SCA for fine-tuning boundary clearances provides a practical advantage in tackling industrial-scale MRLP, as it balances both efficiency and safety considerations.

In terms of computational effort, the proposed IVNS–SCA remains efficient for industrial-scale instances. The IVNS stage requires $\:O\left({n}^{2}\right)$ operations due to pairwise machine moves in the neighborhood structures. The SCA stage adds iterative updates of boundary adjustments, with a complexity of $\:O(n\cdot\:T)$, where $\:n$ is the number of machines and $\:T$ is the number of iterations. Together, these steps yield a polynomial-time algorithm that scales reasonably with problem size. Importantly, the integration of SCA introduces only a negligible overhead: across all 34 instances, the additional runtime was less than one second on average compared to the single-stage counterparts. Even for the largest instances, the overall processing time of the two-stage algorithm was within 300–400 s, which is practical for industrial applications. This demonstrates that IVNS–SCA achieves consistent improvements in layout quality without sacrificing computational efficiency.

Despite these strengths, the scalability of IVNS–SCA is still constrained by the polynomial growth of computational effort, especially in the IVNS stage. For extremely large-scale MRLPs, additional enhancements such as decomposition strategies or parallel computing may be required to maintain tractability. Another promising direction is the development of more efficient MILP models, since exact approaches can guarantee optimal solutions and could serve as a benchmark or hybrid component when combined with metaheuristic methods.

In this study, objectives (1a) and (1b) were integrated into a single-objective formulation. For future research, these two objectives could be treated separately in a multi-objective framework: minimizing material handling cost (objective 1a) and minimizing the penalty from safety distance violations (objective 1b). Such an extension would better capture the potential trade-off between efficiency and safety, where improving one objective may adversely affect the other. In this context, applying a posteriori multi-objective method (such as epsilon constraint, multi-objective metaheuristics) would be more suitable, as they allow decision-makers to explore the Pareto front and select solutions that best fit their preferences and practical constraints.

It is also worth noting that the penalty coefficient β governs the trade-off between flow efficiency and safety compliance: low values risk neglecting safety constraints, while excessively high values may inflate layout size and cost. In our formulation, β is set to balance these two aspects, ensuring that severe hazards (e.g., collisions between machines and operators, or insufficient clearance for fire safety) are heavily penalized, whereas less critical issues such as noise or ergonomic discomfort receive lower penalties. This aligns the optimization results with practical industrial priorities. For future studies, it would be valuable to investigate how β can be calibrated for specific industries or operating environments, since different sectors (e.g., heavy manufacturing, electronics, or food processing) may prioritize safety factors differently, requiring industry-specific values of β to better capture their operational realities.

At last, while this study has compared the proposed IVNS–SCA against established metaheuristics such as SA, GVNS, and their hybrid versions with SCA, future studies could further strengthen the analysis by benchmarking against other widely used or recently developed metaheuristics (e.g., swarm intelligence–based or evolutionary hybrid methods). Incorporating these approaches would provide a broader performance landscape and reveal additional insights into the strengths and limitations of IVNS–SCA. More importantly, the development of new hybrid strategies has the potential to further enhance both the effectiveness of solution quality and the efficiency of computation, offering promising directions for future research.

## Conclusion

A new mathematical model is presented in this study to solve multi row layout problems. The model incorporates safety factors with the objective of minimizing the material handling cost while ensuring the recommended safety distance between machines. A two-stage heuristic is proposed to solve the problem. First, IVNS is employed to allocate the machines in two rows. The subsequent stage involves utilizing the SCA algorithm to optimize the machine placements by establishing the boundary clearance and starting point of each row.

Based on computational experiments on instances of varying complexity, our proposed IVNS + SCA approach outperforms the single stage SA and single stage GVNS based algorithms, as well as the two stage GVNS + SCA method. Furthermore, given that all algorithms have a similar computation time, the IVNS-SCA approach demonstrates superior performance by achieving the best objective value in the majority of instances. On average, it achieved improvements of 0.9% compared to SA–SCA, 1.9% compared to SA, 1.1% compared to IVNS without adjustment, 3.7% compared to GVNS–SCA, and 5.3% compared to GVNS. The advantage was especially evident in large-sized instances, where the boundary clearance adjustment by SCA provided greater opportunities for reducing material handling costs. Although the proposed metaheuristics can deliver promising performance, the search for optimal solutions is still necessary, especially in small instances. Therefore, for the future improvement, it is still important to develop an efficient mathematical model and exact method to solve the problem which can guarantee reaching optimal results in reasonable time.

## Data Availability

The data that support the findings of this study are available from the corresponding author upon reasonable request.
